# Chemical Comparison of White Ginseng before and after Extrusion by UHPLC-Q-Orbitrap-MS/MS and Multivariate Statistical Analysis

**DOI:** 10.1155/2020/4764219

**Published:** 2020-10-07

**Authors:** Yun-Long Guo, Yang Wang, Yi-Lin Zhao, Xiu-Ying Xu, Hao Zhang, Cheng-Bin Zhao, Ming-Zhu Zheng, Shu-Ying Liu, Yu-Zhu Wu, Jing-Sheng Liu

**Affiliations:** ^1^College of Food Science and Engineering, National Engineering Laboratory for Wheat and Corn Deep Processing, Jilin Agricultural University, Changchun 130118, China; ^2^Jilin Ginseng Academy, Changchun University of Chinese Medicine, Changchun 130117, China

## Abstract

Ultrahigh-performance liquid chromatography Quadrupole-Orbitrap tandem mass spectrometry (UHPLC-Q-Orbitrap-MS/MS) was used to compare the composition of ginsenosides in white ginseng (WG) and extruded white ginseng (EWG). A total of 45 saponins, including original neutral ginsenosides, malonyl-ginsenosides, and chemical transformation of ginsenosides, were successfully identified in both WG and EWG. Multivariate statistical analyses including supervised orthogonal partial least squared discrimination analysis (OPLS-DA) and hierarchical clustering analysis (HCA) were used to analyze components of white ginseng before and after extrusion. As a result, three ginsenosides (malonyl (M)-Rb1, M-Rb2, and M-Rc) were found to be increased in WG, while three ginsenosides (Rb2, Rc, and Rg1) were elevated in EWG. In the OPLS-DA S-plot, the different compositions of ginsenoside that were distinguished between WG and EWG were screened out. Experimental results indicate that the UHPLC-Q-Orbitrap-MS/MS is a useful tool to characterize variations of ginsenosides in WG and EWG.

## 1. Introduction

The root of ginseng (*Panax ginseng C. A. Mey.*) has been widely used as traditional medicine and functional foods in China and other Asian countries and regions for over 2,000 years [1]. It is found that ginseng contains lots of bioactive and pharmacological effects ingredients such as polysaccharides, saponin, amino acids, vitamins, protein, and phenolic compounds [2]. In China, WG (white ginseng) and RG (red ginseng) are two main types of ginseng that are widely used in Chinese herbal medicine and food markets. WG is produced by drying fresh ginseng in the sun, and RG is manufactured by steaming fresh ginseng at 95–100°C for 2-3 h and then drying. Although both white ginseng and red ginseng were processed with fresh ginseng, they were used for different purposes in the clinical application of TCM (Traditional Chinese Medicine) practice [3]. Meanwhile, ginseng raw materials and products were popularized worldwide as a natural healthy food along with the global trends of preference for natural products [4]. Modern studies showed that the bioactive and pharmacological effects components are mainly recognized as ginsenosides [5], which exhibit anti-inflammatory, antioxidant, antiapoptotic, anticancer, and immune-stimulant pharmacological activities [6–15].

Extrusion is a continuous process with high temperature, high pressure, and short time. Many chemical and physical reactions occur during the extrusion process, such as starch gelatinization, protein denaturation, and Millard reaction. Meanwhile, functional properties are also changed [16]. Numerous studies have reported that the extruding process could increase some bioactive and pharmacological effects of ginseng products, compared with unprocessed WG [17–19]. The physical characteristics and chemical composition of ginseng are changed during the extrusion process, which further alters the functional properties of ingredients [20–24]. Many researchers have extruded white ginseng and red ginseng samples conducted to improve the chemical and physical properties [8, 16, 18, 19, 25–27]. The contents of crude saponin were higher in the extruded ginseng than that in unprocessed ginseng. In addition, the content of Rg2, Rh1, Rh2, and Rg3 in EWG was increased as the extrusion temperature was raised [28]. WG was more suitable for extrusion than RG because more significant increased antioxidant activity was obtained in extruded WG than that in RG [8]. Although studies on the physical, chemical, and functional properties of extrusion WG have been reported in the literature, the comparison of ginsenoside composition of WG before and after extrusion process has not yet been researched.

In the present study, we applied UHPLC-Q-Orbitrap-MS/MS combined with multivariate statistical analysis approach to assess the ginsenoside compositions of WG and EWG. We also investigated the changing chemical structures of WG and EWG and the possible reasons. 

## 2. Materials and Methods

### 2.1. Materials and Reagents

HPLC-MS grade methanol, acetonitrile, and formic acid were purchased from TEDIA (Fairfield, OH, USA). Ultrapure water was filtered through a Milli-Q system (Millipore, Billerica, MA, USA). All other chemical reagents were of analytical grade. Ginsenoside Rb1, ginsenoside Rb2, ginsenoside Rb3, ginsenoside Rc, ginsenoside Rd, ginsenoside Re, ginsenoside Rf, ginsenoside Rg1, ginsenoside Rg2, ginsenoside Ro, and standard chemicals were purchased from Shanghai Standard Technology Co., Ltd. (purity ≥ 98%, Shanghai, China). The 5-year-old white ginseng power was obtained from Hongjiu Biotech Co., Ltd. (total saponins of Panax ginseng was 4.5%, moisture was 4.8%, Jilin, China).

### 2.2. Extrusion Process

The WG root powder was extruded with a co-rotating intermeshing twin-screw extruder (Fumach Food Stuff Engineering & Technology Co., Hunan, China). The parameters of extrusion were as follows: the moisture content of 20%, screw speed of 200 rpm, the feed rate of 10 kg/h, and die diameter of 3.0 mm. The temperature profile from the feed section to the die exit was set to 140°C. The extrudate was directly dried in the oven at 60°C for 12 h.

### 2.3. Extraction of Ginsenosides

The obtained powder was weighed (0.1 g) and extracted with 5 mL of 70% methanol in an ultrasonic water bath for 45 min, and the extract was filtered through a syringe filter (0.22 *μ*m) and stored in a 4°C refrigerator for LC-MS analysis [29, 30].

### 2.4. The Methods of UHPLC-Q-Orbitrap-MS/MS Analysis

Chromatographic separation was performed on an Ultimate 3000 ultrahigh-performance liquid chromatography system (Thermo Fisher, San Jose, CA, USA) coupled with the Supelco C_18_ column (3.0 × 50 mm, 2.7 *μ*m; Sigma-Aldrich, USA). The column oven temperature was maintained at 35°C, and the mobile phases A and B were acetonitrile and water with 0.1% formic acid, respectively. The separation of experimental samples was programmed with the following gradient elution: the proportion of acetonitrile (A) was increased from 15% to 19% (0–5 min), 19–19% (5–10 min), 19–25% (10–13 min), 25–28% (13–15 min), 28–28% (15–18 min), 28–30% (18–22 min), 30–35% (22–25 min), 35–40% (25–30 min), 40–60% (30–35 min), 60–80% (35–38 min), 80–100% (38–40 min), 100–100% (40–45 min), and finally adjusted from 100% to 15% (45–50 min) and maintained at 15% for 10 minutes. The injection volume was 5 *μ*L, and the flow rate was 0.4 mL/min.

Mass spectrometric detection was carried out on a Q-Orbitrap-MS/MS (Thermo Fisher, San Jose, CA, USA) equipped with electrospray ionization source operated in the negative ion mode. The parameters of ion source were set as follows: sheath gas flow of 35 Arb, aux gas flow of 10 Arb, and sweep gas flow of 1 Arb. S-Lens RF was 55%. The capillary voltage was set to −3.5 kV with a capillary temperature of 350°C. Full-scan MS data were acquired at the centroid mode from m/z 150 to 2000 Da, 70,000 resolution, automatic gain control (AGC), the target of 1 × 10^6^, and maximum injection time (IT) of 100 ms. The parameters of dd-MS2 were set as follows: 17,000 resolution, automatic gain control (AGC) the target of 1 × 10^5^, maximum injection time (IT) of 50 ms, Loop count 5, isolation window 4.0 m/z and NCE/stepped NCE.The MS/MS data were acquired in Full-MS/ddMS2 mode using the following settings: resolution 17,000 with AGC target of 1×105, maximum IT of 50 ms, and the normalized collision energy (NCE) of 25–55.

### 2.5. Data Processing and Multivariate Analysis

The SIEVE (version2.1, Thermo Fisher, San Jose, CA, USA) software was used to process the raw data of samples, which could detect the mass, retention time, and intensity of the peaks in each TIC. The max retention time shift was set at 0.20 min, and the m/z width was 10 ppm to align the features. The base peak min intensity and background were set at 10^5^ and 3, respectively. After being aligned, the intensity of each ion was normalized by the total ion intensity of each TIC. The resultant dataset, containing m/z value @ retention time, the normalized intensity, and the sample code, was used to perform the multivariate statistical analysis. Then, the datasets were saved as .csv files and imported into SIMCA-P software 11.5 (Umetrics, Umea, Sweden) to conduct the multivariate statistical analysis, including orthogonal partial least squared discrimination analysis (OPLS-DA) and hierarchical clustering analysis (HCA). In the OPLS-DA model, ions with variable importance in projection VIP values larger than 1 were highlighted and were further filtered by *t*-test (SPSS19.0, Chicago, IL, USA). The components with *p* < 0.05 were considered significant and were selected as analytical markers.

## 3. Results and Discussion

### 3.1. UHPLC-Q-Orbitrap-MS/MS Analysis of White Ginseng and Extruded White Ginseng

**Figure 1:**
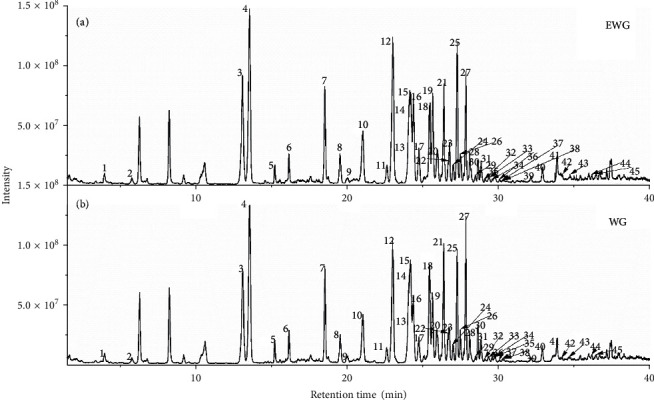
Total ion chromatogram (TIC) of extruded white ginseng (a) and white ginseng (b).

The ultrahigh-performance liquid chromatography combined high-resolution mass spectrometry has been proved as an effective analytical tool for ginsenoside analysis in complex extracts of Chinese herb medicine [31–34].  Figure 1 shows the total ion chromatogram (TIC) of the extracts of WG and EWG samples by UHPLC-Q-Orbitrap/MS in the negative ion mode. The ginsenoside compounds were effectively separated in 45 min by the established method. The intensities of several peaks were different before and after extrusion. Compared to WG, the total ion chromatogram of EWG showed obvious lower or higher intensity of some peaks from 25 to 30 min. These demonstrated that the extrusion process changed the chemical composition of the ginseng sample. Meanwhile, the sample of ginseng contains water, and the determination of water content may be performed before extraction [35, 36], which contribute to accurate determination of polysaccharide in future research.

The Q-Orbitrap-MS was reliable and sensitive for measuring the exact mass values of the compounds from samples. Moreover, the retention time, accurate masses, and the characteristic fragment ions were compared with the standards to perform the compound identification.

**Figure 2:**
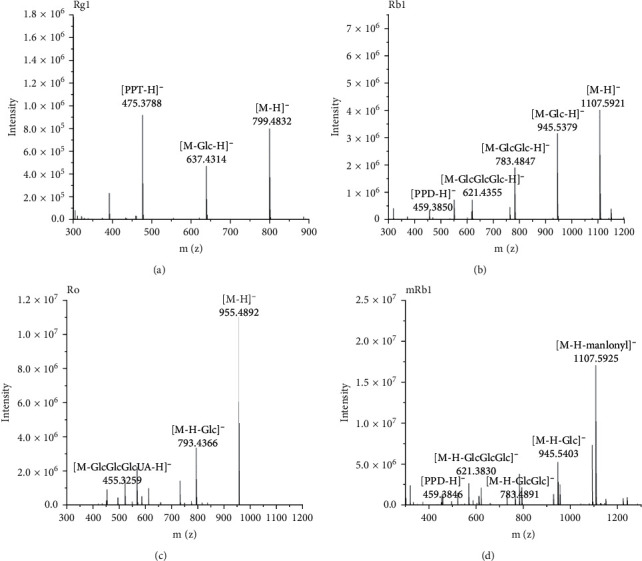
The tandem mass spectrum of ginsenosides in the negative ion mode: (a) Rg1; (b) Rb1; (c) Ro; (d) mRb1.

Because the negative ion mode gave a much clearer fragmentation pattern for structure identification of ginsenoside compounds, we detected ginsenoside and confirmed the molecular weight by full-scan MS in negative ion mode. The [M-H]^−^ and [M + HCOO]^−^ ions of ginsenoside were formed in the negative ion mode. Compared to the theoretical value, all molecular ions were measured within the mass accuracy of 10 ppm.  Table 1 shows the characteristic fragment ions of ginsenosides. The aglycone type and the sequence of ginsenosides were confirmed by the tandem MS information.  Figure 2 shows the tandem MS spectra of protopanaxadiol saponins (Rg1 and Rb1), malonyl saponins (mRb1), and oleanolic acid saponins (Ro). As shown in Figures 2(a)–2(c), the [M-H]^−^ ion produced fragment ions at m/z 475, 459, and 455, which were the characteristic ions of ginsenosides. The fragment ion at m/z 475 was produced by loss of two glucose residues (162 Da + 162 Da). The fragment ion at m/z 459 was subtracted from four glucose residues (162 Da + 162 Da + 162 Da + 162 Da). Figures 2(c) and 2(d) show the MS/MS spectra of ginsenoside Ro and malonyl-ginsenoside Rb1. The fragment ion at m/z 455 was produced by loss of two glucose residues and *β*-d-glucuronic acid (162 Da + 162 Da + 176 Da). In Figure 2(d), the [M-H]^−^ ion produced fragment ions at m/z 1107, representing malonyl-ginsenoside cleavage by loss of one malonyl residue. Similar to Rb1, the fragment ion at m/z 459 is produced by loss of four glucose residues (162 Da + 162 Da + 162 Da + 162 Da). The experimental results show that the tandem mass spectrum fragmentation provided structural rich information for the elucidation of ginsenosides. By comparing the retention time, full scan, and the tandem MS spectra with the standard, ginsenoside structure could be identified. The components without standard could be tentatively assigned by comparing the data with the literature record. As a result of our analysis, a total of 45 ginsenosides were identified from WG and EWG. The identification information of ginsenosides is listed in Table 1.

### 3.2. Multivariate Statistical Analysis of White Ginseng and Extruded White Ginseng

Some researchers obtained chemical profiling data using high-resolution mass spectrometry combined with multivariate statistical analysis [37–41]. The antioxidant activity of EWG was better than that of WG because of the chemical composition changes after extruding [16, 18]. Thus, we used statistical methods to explore the differences in composition. The extract of WG and EWG was analyzed by the UHPLC-Q-Orbitrap-MS/MS. After data preprocessing, the dataset was generated and used to conduct multivariate statistical analysis.

**Figure 3:**
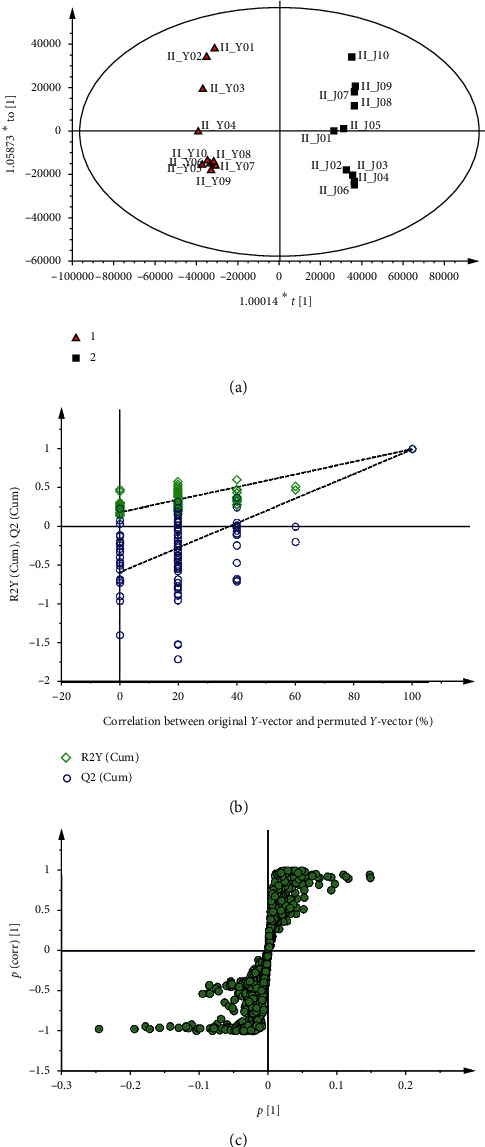
The OPLS-DA/permutation test/S-plot of white ginseng and extruded white ginseng in negative ion mode.

Multivariate data analyses were performed to characterize the distinct composition of various chemicals from WG and EWG in detail. As shown in Figure 3(a), the score plot of OPLS-DA were effectively used todistinguishbetween WG and EWG samples. No overfitting was found because the permutation *R*^2^ (0.993) and *Q*^2^ (0.989) values on the right are higher than those on the left (Figure 3(b)). In the S-plot, the points of the chemical composition that importantly contributed to the variance between WG and EWG were plotted farther along the *x*-axis and *y*-axis (Figure 3(c)). In the comparison between WG and EWG, the increased components of WG were shown in the lower left quadrant of S-plot, while the upper right quadrant showed the increased components of EWG. The results showed that three ginsenosides (malonyl (M)-Rb1, M-Rb2, and M-Rc) and three ginsenosides (Rb2, Rc, and Rg1) were elevated in WG and EWG, respectively. In the OPLS-DA and S-plot, different compositions of the chemical were distinguished between WG and EWG.

**Figure 4:**
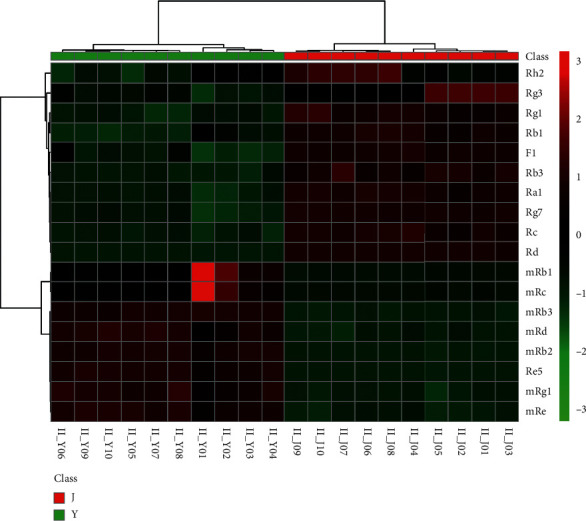
The heat maps visualizing the intensities of the 17 ginsenoside datasets from white ginseng and extruded white ginseng (*n* = 10) and extruded white ginseng (*n* = 10).

In order to visualize the tendency of the variation of the chemical markers of WG and EWG, a heat map was constructed based on the relative intensity of each compound. As shown in Figure 4, color differences indicated the composition change in the WG group and the EWG group. Between the WG and EWG groups , the contents ofginsenoside-Rh2, -Rg3, -Rg1, -F1, -Rb3, -Ra1, -Rg7, -Rc, and Rd in EWG samples were significantly higher. However, the contents of malonylginsenoside-Rb1, -Rc, -Rb3, -Rd, -Rb2, -Rg1, and -Re, and ginsenoside-Re5 in all the WG samples were significantly higher.. This was illustrated in the heating trial, in which the concentration of ginsenosides was affected by the thermal processing condition and the degree of conversion between malonyl and neutral ginsenosides [37, 42, 43]. The results suggested that malonyl-ginsenoside was thermally unstable and especially susceptible to hydrolysis by extruding treatment. The malonyl residue was well preserved in WG because its dehydration was conducted in sunlight without extruding treatment.

In the case of crude saponin (some ginsenosides such as Rb1, Rg1, and Rc) and rare saponin (some ginsenosides such as Rh2 and Rg3) content, there was a slight increase after extrusion. The malonyl-ginsenoside is prone to be transformed into ginsenoside due to heat and pressure treatment; the conversion efficiency of the ginsenosides was increased by the extrusion process. The amount of several ginsenosides was increased by the extrusion process because the cell or tissues of ginseng underwent transformations, which allowed easier extraction of these components through the extrusion process. Meanwhile, this result was caused by the weakening of molecular bonds and increased water absorption at the high temperature, pressure, and shear forces involved in the extrusion process.

## 4. Conclusions

In this study, we perform the chemical comparison of white ginseng before and after extrusion by UHPLC-Q-Orbitrap-MS/MS combined with multivariate statistical analysis. Multivariate statistical analyses were applied to differentiate the chemical components of WG and EWG, and 6 ginsenosides were found as the characteristic chemical markers of the analytical sample. The experimental information will help to supervise the production of each type of processed ginseng product in the herbal medicine market or food industry. Finally, this study verified the application value of this method in the quality evaluation of WG and EWG. It is expected that the established method will be useful for evaluating processed products not only by ginseng samples but also other agricultural and food product processing such as tea, grape, meat, and so on.

## Figures and Tables

**Table 1:** Compounds identified from white ginseng and extruded white ginseng.

No.	Rt (min)	Formula	Detected mass (Da)	Mass error (ppm)	MS/MS fragment ions	Identification
1	3.37	C_42_H_72_O_15_	861.4844^a^	1.0	650.2734, 542.4258, 415.3136	Ginsenoside Re_5_
2	5.81	C_22_H_32_O_13_	503.1771^b^	0.2	473.1600, 415.2855, 390.2661	Cistanoside H
3	13.14	C_42_H_72_O_14_	845.4894^a^	1.2	799.4832, 637.4314, 475.3788	Ginsenoside Rg_1_
4	13.57	C_48_H_82_O_18_	991.5472^a^	1.1	945.5403, 799.4826, 637.4302, 475.3785	Ginsenoside Re
5	15.24	C_45_H_74_O_17_	885.4991^b^	1.4	841.4911, 781.4704, 637.4300, 475.3782	Malonyl-ginsenoside Rg_1_
6	16.2	C_51_H_84_O_21_	1031.5425^b^	0.7	945.5348, 783.4891, 637.4325, 475.3785	Malonyl-ginsenoside Re
7	18.55	C_42_H_72_O_14_	845.4893^a^	1.3	799.4816, 637.4309, 475.3787	Ginsenoside Rf
8	19.57	C_41_H_70_O_13_	815.4793^a^	0.6	637.4122,475.2591	Noto-ginsenoside R_2_
9	20.04	C_59_H_100_O_27_	1239.6353^b^	0.9	1077.5878, 945.5434, 783.4840, 681.4325	Noto-ginsenoside R4/ginsenoside Ra3
10	21.03	C_42_H_72_O_13_	829.4858^a^	0.4	637.4292, 475.3785	Ginsenoside Rg_2_
11	22.64	C_58_H_98_O_26_	1255.6304^a^	2.2	1239.6354, 1209.6183, 1007.5946, 945.5434, 799.4820, 650.1565, 475.3796	Ginsenoside Ra_2_
12	23.04	C_54_H_92_O_23_	1153.5977^a^	1.2	1107.5927,945.5379,783.4874, 621.4355, 459.3850	Ginsenoside Rb_1_
13	24.06	C_48_H_76_O_19_	955.4892^b^	1.7	793.4366, 613.3745, 569.3832, 455.3529	Ginsenoside Ro
14	24.19	C_57_H_94_O_26_	1193.5942^b^	1.6	1107.5925, 945.5403, 783.4891, 621.3830, 459.3846	Malonyl-ginsenoside Rb_1_/isomer
15	24.39	C_53_H_90_O_22_	1123.5885^a^	1.9	1077.5790, 945.5386,7 83.4573, 621.2311,459.3891	Ginsenoside Rc
16	24.75	C_58_H_98_O_26_	1255.6209^a^	1.5	1239.6340, 1209.6176, 1007.5927, 945.5438, 799.4827, 650.1566, 475.3743	Ginsenoside Ra_1_
17	25.25	C_36_H_62_O_9_	683.4382^a^	0.9	475.3733	Ginsenoside F_1_
18	25.46	C_56_H_92_O_25_	1163.5841^b^	1.2	1077.5323,945.5419,783.4745, 621.42631,459.3257	Malonyl-ginsenoside Rc
19	25.66	C_53_H_90_O_22_	1123.5887^a^	1.7	1077.5820, 945.5396, 783.4877, 621.4354, 459.3839	Ginsenoside Rb_2_
20	25.95	C_57_H_94_O_26_	1193.5946^b^	1.3	1149.6058, 1177.5963, 945.5458, 783.4924, 621.4364, 459.3817	Malonyl-ginsenoside Rb_1_/isomer
21	26.03	C_53_H_90_O_22_	1123.5873^a^	2.9	1077.5817, 945.5402, 783.4875, 621.4353, 459.3840	Ginsenoside Rb_3_
22	26.4	C_56_H_92_O_25_	1163.584^b^	1.3	1077.5225,945.5573,783.4354, 621.4602, 459.3924	Malonyl-ginsenoside Rb_2_/isomer
23	26.75	C_56_H_92_O_25_	1163.584^b^	1.3	1077.5347, 945.5523, 783.4616, 621.4473, 459.3248	Malonyl-ginsenoside Rb_2_/isomer
24	27.02	C_42_H_66_O_14_	793.4374^b^	0.8	631.3840, 569.3831, 455.3528	Chikusetsusaponin IVa
25	27.27	C_48_H_82_O_18_	991.5472^a^	1.5	783.4174, 621.3566, 459.3527	Ginsenoside Rd
26	27.48	C_56_H_92_O_25_	1163.5837^b^	1.5	1077.5360, 945.5741, 783.4784, 621.4541	Malonyl-ginsenoside Rb_3_/isomer
27	27.83	C_51_H_84_O_21_	1031.5424^b^	0.8	945.5354, 783.4422, 637.4179, 475.3627	Malonyl-ginsenoside Rd
28	28.1	C_50_H_84_O_19_	1033.5565^a^	2.3	945.2658, 783.2891, 637.4630, 475.2571	Acetylginsenoside Re
29	28.22	C_55_H_92_O_23_	1165.6007^a^	0.3	1077.5847, 945.5405, 783.4880, 621.4357, 459.3843	GinsenosideRs1/Rs2/isomer
30	28.65	C_55_H_92_O_23_	1119.5957^b^	0.5	1077.5479, 945.5371, 783.4766, 621.4918, 459.3737	Ginsenoside Rs1/Rs2/isomer
31	28.86	C_51_H_84_O_21_	1031.5422^b^	0.1	945.52647, 783.4873, 621.4560, 459.7781	Malonyl-ginsenoside Rd/isomer
32	29.11	C_51_H_84_O_21_	1031.5421^b^	0.2	945.5331, 783.4842, 621.4179, 459.3679	Malonyl-ginsenoside Rd/isomer
33	29.34	C_54_H_86_O_24_	1117.5417^b^	1.7	945.5157, 783.4358, 621.4479, 459.3297	PPD-Glc-(Glc-Glc)-2malonyl
34	29.41	C_47_H_80_O_17_	961.8364^a^	1.5	915.5361, 785.9601, 621.4492, 459.3247	Noto-ginsenoside Fe/vinaginsenoside R_16_
35	29.58	C_54_H_86_O_24_	1117.5422^b^	1.3	945.5359, 927.6159, 783.4793, 621.6795	PPD-Glc-(Glc-Glc)-2malonyl
36	29.77	C_50_H_84_O_19_	1033.5579^a^	1.0	987.5502,945.5998,783.4875, 459.3838	Pseudo-ginsenoside Rc_1_
37	30.08	C_47_H_80_O_17_	961.5368^a^	1.0	915.5049, 783.2958, 621.4138, 459.3660	Noto-ginsenoside Fe/vinaginsenoside R_16_
38	30.26	C_50_H_82_O_20_	1001.5317^b^	1.0	915.5296, 783.4793, 622.4159, 459.3647	Malonylnoto-ginsenoside Fe
39	32.16	C_42_H_72_O_13_	829.4955^a^	0.8	621.42671, 459.3097	Ginsenoside F_2_
40	32.89	C_42_H_66_O_14_	793.4374^b^	0.8	631.3840, 569.3831, 497.3645, 455.3528	Zingibroside R_1_
41	33.86	C_42_H_72_O_13_	829.4947^a^	1.0	637.4154, 475.3612	Ginsenoside Rg_3_/isomer
42	34.12	C_42_H_72_O_13_	829.4946^a^	1.1	637.4304,475.3143	Ginsenoside Rg_3_/isomer
43	34.72	C_42_H_72_O_14_	799.4841^b^	0.8	734.8464, 621.4331, 542.4661, 481.0309	Ginsenoside Rg_7_
44	36.44	C_42_H_70_O_12_	811.4843^a^	0.7	765.4680, 649.9458, 545.0465, 432.8917, 304.9154	Ginsenoside Rg_5_
45	36.70	C_36_H_62_O_8_	667.4419^a^	1.2	631.3836, 455.5328, 304.9154	Rh_2_

^a^[M+COOH]^−^;  ^b^[M − H]^−^.

## Data Availability

The data used to support the results of this study are included within the article. Any further information is available from the authors upon request.
